# Supplementary material for: Causal Relationship between Aging and Anorexia Nervosa: A White-Matter-Microstructure-Mediated Mendelian Randomization Analysis
Source: Biomedicines. 2024 Aug 16;12(8):1874. doi: 10.3390/biomedicines12081874 (PMC11351342; doi:10.3390/biomedicines12081874)
Supplement: Supplementary file 1 [file biomedicines-12-01874-s001.zip › Supplementary Figure/Figure S8/Leaveoneout plot of MR analysis for TL to ubm-b-1940.pdf]

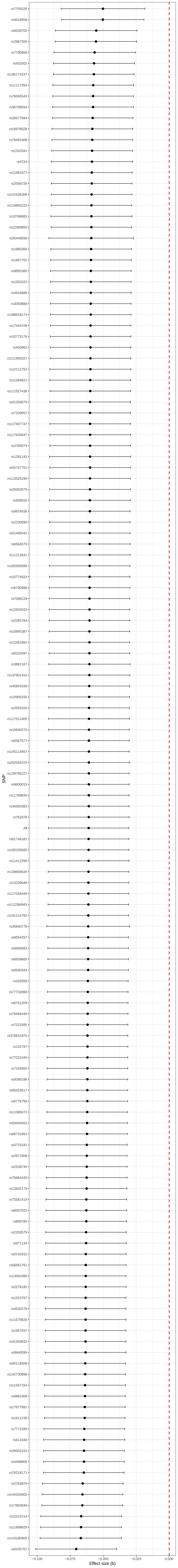
rs7705526
rs9419958
rs6536702
rs2967355
rs7790856
rs932002
rs185174247
rs11117354
rs76065543
rs56799554
rs28577594
rs16978028
rs78491606
rs1332941
rs4724
rs11991877
rs2056726
rs142426306
rs116863223
rs10768683
rs12369950
rs35446936
rs1985369
rs1907702
rs9955360
rs1003322
rs4616688
rs3093888
rs188918174
rs17445108
rs10773176
rs450962
rs111950327
rs10112752
rs11584821
rs111527438
rs41269079
rs7209057
rs117407747
rs117630647
rs3785074
rs1291143
rs55747751
rs113525195
rs28363070
rs939916
rs9878436
rs2230590
rs61405042
rs6584579
rs11212631
rs182059586
rs10774624
rs6790988
rs7099229
rs12925933
rs2282764
rs10845387
rs12451892
rs8102497
rs3891167
rs137901416
rs45604339
rs10905255
rs2555104
rs117512405
rs10840270
rs6587577
rs145114957
rs202034370
rs139795227
rs9600019
rs11769630
rs34550383
rs762679
All
rs61748181
rs150150565
rs11412296
rs139669835
rs13230646
rs117034449
rs112394943
rs141214782
rs35640778
rs6054257
rs6669563
rs6659669
rs6590343
rs429358
rs77732866
rs6751209
rs76666449
rs7221585
rs376641875
rs131797
rs77231040
rs7164950
rs9398196
rs80324517
rs6776756
rs11085072
rs59409453
rs66731853
rs4731541
rs2977608
rs2538745
rs75664430
rs12932179
rs73581419
rs6007020
rs869785
rs2293579
rs871134
rs5742915
rs56061761
rs13062095
rs2276182
rs1023767
rs4530278
rs11579626
rs1957937
rs41304832
rs9940099
rs80116508
rs142730696
rs11557154
rs6881568
rs17677991
rs1611236
rs7772289
rs611646
rs28502153
rs4498805
rs76219171
rs2763979
rs144204502
rs17803849
rs12412214
rs11699829
rs143190905
rs8105767
SNP
−0.100
−0.075
−0.050
−0.025
0.000
Effect size (b)
